# Amino Acid-Based Hydrogel with Interpenetrating Gelatin and Cross-Linked by Metal Ions, Providing High Stretchability and Motion Sensitivity

**DOI:** 10.1021/acsomega.4c10083

**Published:** 2025-03-17

**Authors:** Samaneh Khodami, Klaudia Kaniewska, Jan Romanski, Marcin Karbarz, Zbigniew Stojek

**Affiliations:** † University of Warsaw, Faculty of Chemistry, 1 Pasteura Str., Warsaw 02-093, Poland; ‡ Biological and Chemical Research Center, University of Warsaw, 101 Żwirki I Wigury Av., PL, Warsaw 02-089, Poland

## Abstract

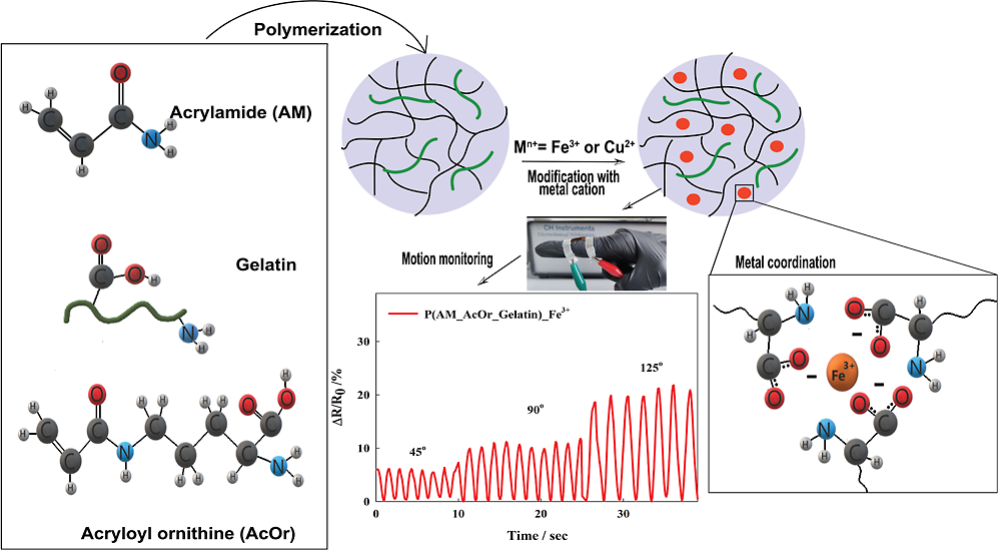

A double network structure with metal ions was created to enhance the mechanical stability of the hydrogels and increase their low conductivity. For this purpose, the P(AM_AcOr_Gelatin) hydrogel was synthesized by combining gelatin, a biocompatible polymer, *N*-δ-acryloyl-ornithine (AcOr), an amino acid derivative, and acrylamide (AM). Because the amino acid-based monomer added charged groups to the hydrogel network, the hydrogel exhibited improved conductivity and motion sensitivity properties compared with polyacrylamide (PAM) hydrogels. Furthermore, we altered the P(AM_AcOr_Gelatin) hydrogel by introducing the Fe^3+^ and Cu^2+^ ions, resulting in the formation of the P(AM_AcOr_Gelatin)-Fe^3+^ and P(AM_AcOr_Gelatin)-Cu^2+^ hydrogels. The hydrogels containing metal ions had coordination bonds between the ions, gelatin, and AcOr. Additionally, there were other noncovalent bonds present, resulting in further increased conductivity (approximately 95% improvement) and stretchability (more than double). The conductivity and resistance of the hydrogels changed, depending on the bending position and strain applied to the hydrogel layer. The results demonstrated that the hydrogel layer had good strain sensitivity, with an enhanced gauge factor (GF) of approximately 1.7 (at 250% strain) and a conductivity ranging from 3355 to 4387 μS·cm^–1^.

## 1 Introduction

Hydrogels have attracted considerable attention because of their unique properties, including their high water content, flexibility, and sensitivity to parameters like pH, temperature, and strain. The increasing number of hydrogel applications has led to efforts to develop simpler production methods for new and enhanced hydrogel formulations.^1,2^

Hydrogels are excellent materials for making sensitive and adaptive sensors.^3−7^ One of the challenges that still remains is to achieve stability, high sensitivity, and exceptional stretchability when designing these hydrogels for use as dressings and wearable sensors.^8−11^ These hydrogels are designed to respond to mechanical stimuli, such as pressure, stretching, and bending, by undergoing reversible changes in their structure and properties. Researchers have integrated various responsive components with the hydrogel matrix to create materials that function as suitable sensors. The adaptability of these hydrogels makes them appropriate for diverse applications, including artificial tissue, biomedical implants, and soft robotics.^12−14^ The distinctive properties of stretchable and conductive hydrogels open doors to innovative applications. In wearable technology, they may be seamlessly integrated into garments and devices to monitor human movement accurately and reliably. Furthermore, in soft robotics, these hydrogels may serve as ideal materials for constructing artificial muscles, enabling more lifelike interactions with the environment. Additionally, in biomedical engineering, they hold promise for next-generation prosthetics and implants that seamlessly integrate with body movements while providing real-time physiological feedback. In essence, the remarkable stretchability and conductivity of these hydrogels pave the way for transformative advancements in various fields, from healthcare to wearable electronics.^15−18^

Researchers have developed sensors using both synthesized and natural polymers such as gelatin, alginate, and silks.^19,20^ However, there are some challenges with hydrogel-based sensors such as low stretchability and poor long-term stability. These issues make strain sensors unreliable for prolonged use and hinder their ability to detect subtle changes in movement monitoring.^21−23^ Due to their poor conductivity, hydrogels have limited prospects in strain sensors and biosensors. To address this issue, scientists have developed various methods to increase the conductivity of hydrogels. For example, they have incorporated channels for charge transport, as well as conductive nanoparticles like graphene, carbon nanotubes, and metal ions into the hydrogel matrix to enhance its electrical characteristics.^24,25^ Hydrogels can also be made more conductive by adding functional groups that facilitate ion transport or by doping them with conductive polymers. This will allow hydrogels to be used in more sophisticated applications that require electrical conductivity.^25,26^

To compare strain sensitivity, the relative electrical resistance change of strain sensors when they are deformed was measured, which is known as the gauge factor (GF). Typically, strain sensors made from hydrogels show lower GF values compared to those made from elastomers.^27^ This makes it challenging to achieve high sensitivity in hydrogel-based strain sensors. However, the porous structure and high water content of hydrogels allow for easy deformation under strain, which restricts ion movement and ultimately results in good strain sensitivity.

For example, Liu and colleagues synthesized a piezoresistive hydrogel with enough ferric ions in the hydrogel network. They demonstrated a gauge factor of 0.5 at 200% strain.^28^ In a related study, Zhou et al.^29^ added NaCl in the hydrogels to improve ion conductivity to 2.6 S·m^–1^, but they reduced the GF to below 0.75. However, Cai and others^30^ incorporated single-wall carbon nanotubes (SWCNTs) into the hydrogel matrix, improving the strain sensitivity of the hydrogels from a GF of 0.5 to GF = 1.5 (at 1000% strain).

On the other hand, hydrogel-based sensors need to have stretchability, toughness, and durability to withstand dynamic and recurring loading in practical applications. However, many hydrogels have mechanical weaknesses such as low toughness and moderate stretchability.^31^ He and his team developed a durable and conductive strain sensor using a specially designed double-network hydrogel.^32^ The mechanical properties were enhanced by the interaction of chemical bonds in the double network, coordination bonds, and hydrogen bonds, resulting in exceptional mechanical performance such as stretchability of over 2000% and toughness of up to 38.8 kJ·m^2^.

The double-network hydrogels are somewhat unique.^33−37^ Gong et al. first showed that the double network structure of the hydrogel makes the gel tough and stretchable.^38^ The second interpenetrating network is usually made up of a neutral or polyelectrolyte polymer. To get remarkable mechanical properties of the hydrogel, the first network is usually a rigid polyelectrolyte, and the second one is a flexible neutral polymer. Traditional hydrogels exhibit poor mechanical stability, and under force, the first-network molecular chains break, causing significant gel softening and low fatigue resistance. To address these challenges, the incorporation of recoverable physical bonds, as additional cross-links, into the network proves to be effective. For example, the introduction of gelatin as the second network can improve the toughness of the hydrogels.^39−41^ Gelatin has excellent biocompatibility and so is used in drug delivery, pharmaceuticals, and food industry. It has several active groups: amine, pyridine, and hydroxyl. Because of its qualities, gelatin becomes a useful ingredient in the construction of better double-network hydrogels.^42,43^

Therefore, we designed a double-network hydrogel incorporating gelatin in this research. The first network was created via copolymerization between acrylamide and amino acid-based monomer.

Amino acids are crucial molecules in living organisms, because they are essential components of organic synthesis, particularly as chiral auxiliaries and building blocks. Researchers, in a variety of domains, investigated stimulus-responsive polymer networks based on amino acids. The potential use of these responsive polymers in drug delivery, artificial muscles, separation methods, enzyme immobilization, sensors, shock absorbers, and molecular recognition has drawn much attention.^44,45^ Additionally, amino acids, readily available in nature and easily synthesized, constitute fundamental building blocks of proteins.^46^ In aquatic environments, compounds based on amino acids can engage in diverse noncovalent interactions, encompassing hydrogen bonding, electrostatic interactions, and hydrophobic interactions. Additionally, amino acids exhibit significant water solubility, biocompatibility, and degradability, along with the capacity to influence polymer conformation.^47,48^ The incorporation of amino acid moieties into gels can impart such properties, such as sensitivity to pH, ionic strength, and specific ions. Gel polymeric materials for biomedical and industrial purposes may include amino acid residues in the main chain, because they aim at biocompatibility and biodegradability similar to polypeptides.^49−51^ Also, ornithine (and its derivative acryloyl ornithine) is important and competitive among other chemical groups, as it serves as one of the fundamental building blocks in living organisms.

The mostly unexplored area of amino acid hydrogels with coordinated metal ions has provided a novel aspect in the development of smart materials. The interaction between the metal and ligand in hydrogels improves binding strength in sensing units and promotes resistance to fracture stress. Furthermore, the integration of metal ions improves conductivity, enabling applications like motion sensors and electronic skins.^52−55^

Regarding the interactions with metal cations, ornithine is rather a strong ligand. Ferric and copper ions are two options for the creation of metal complexes with amino acids. Logarithms of stability constants for Fe(III) species consistently fall within the 8–10 range, whereas in the case of Cu(II)–amino acid complexes, logarithms of stability constants equal from 8 to 14. The Fe(II) complexes with amino acids are notably weaker than those observed for Cu(II), and, in general, the stability constants of amino acid complexes of Cu(II) surpass those of Cu(I).^56,57^

In the present study, we synthesized the amino-acid-based p(AM_AcOr_Gelatin) hydrogel through a one-step copolymerization strategy involving acrylamide (AM), acryloyl ornithine (AcOr), and gelatin. Inspired by our previous research^58^ and the examination of the constants of metal–ligand complexes,^59,60^ we introduced the Cu^2+^ and Fe^3+^ ions into the P(AM_AcOr_Gelatin) hydrogel and created the P(AM_AcOr_Gelatin)-Cu^2+^ and -Fe^3+^ hydrogels where metal ions were coordinated by gelatin and AcOr. In contrast to traditional PAM hydrogels, the incorporation of AcOr and gelatin, as the second network, facilitated physical cross-linking via multiple hydrogen and electrostatic bonds. Finally, the P(AM_AcOr_Gelatin) hydrogel exhibited a high degree of stretchability and stability. Moreover, compared to the P(AM_AcOr_Gelatin) hydrogel, solely cross-linked through hydrogen and electrostatic bonding, the P(AM_AcOr_Gelatin)-Cu^2+^ and -Fe^3+^ hydrogels featured a dual cross-linked structure with additional AcOr–Cu^2+^ and AcOr–Fe^3+^ coordination bonds. This led to improved mechanical properties and required conductivity.

Apparently, the P(AM_AcOr_Gelatin)-Cu^2+^ and -Fe^3+^ hydrogels should be promising materials for wearable strain sensors and should exhibit significant resistance to deformation. The urgent need for flexible strain sensors is seen in such fields as robotics, motion detection, actuators, aerospace, and electronic skins.

## 2 Experimental Part

### 2.1 Materials

AM, gelatin from bovine skin (gel strength −225 g Bloom, Type B), *N*,*N*′-methylenebis (acrylamide) (BIS, >99%), ammonium persulfate (APS, >98%), and *N*,*N*,*N*′,*N*′-tetramethylethylenediamine (TEMED) were purchased from Sigma-Aldrich. FeCl_3_·6H_2_O was provided by Aldrich and Cu(NO_3_)_2_·3H_2_O by the ACROS company. AcOr was synthesized by our group and characterized in one of our previous papers.^61^ All compounds were utilized in their original state. The solutions were prepared by using high-purity water from a Milli-Q Plus/Millipore purification system (water conductivity: 0.05 μS·cm^–1^).

### 2.2 Synthesis and Modification of P(AM_AcOr_Gelatin) Hydrogels

P(AM_AcOr_Gelatin) hydrogels were obtained by using the free-radical polymerization method. The overall monomer concentration was 1 M; the contributions of AM, AcOr, and BIS were 94, 5, and 1 mol %, respectively. The concentrations of APS and TEMED were 2.0 mM (0.2 mol % vs total concentration) and 33 mM (3.3 mol % vs total concentration), respectively. The gelatin quantity was 10 g·L^–1^. The polymerization occurred in three sequential steps. Initially, gelatin was dissolved in water at 40 °C. Next, the AM, AcOr, and BIS monomers were introduced, and the mixture was stirred for 20 min under a nitrogen atmosphere at room temperature. Finally, the APS solution and TEMED were swiftly added. The resulting solution was poured into a designed mold consisting of two parallel glass pieces separated by a 1 mm silicon-sealed gap. The polymerization commenced at room temperature upon the addition of APS and proceeded overnight. The P(AM_AcOr_Gelatin)-Fe^3+^ and -Cu^2+^ hydrogels were prepared by adding 100 μL of 1 M FeCl_3_ and Cu(NO_3_)_2_ aqua solutions to 300 μL of the P(AM_AcOr_Gelatin) hydrogels.

Furthermore, samples P(AM_AcOr) and P(AM_Gelatin), which lacked the AcOr monomer in the structure and gelatin, respectively, were prepared for comparison purpose.

### 2.3 ^1^H NMR and FT-IR Measurements

^1^HNMR spectra were acquired in deuterium oxide at 22 °C with a Bruker 300 MHz spectrometer. The samples were freeze-dried. Subsequently, the samples were crushed and immersed in deuterium oxide (D_2_O).

The FT-IR spectra of dried samples were recorded by using a Thermo Scientific Nicolet iS50 spectrometer.

### 2.4 Swelling Ratio and Water Content

To determine the swelling ratio and water content percentage, we weighed the dry hydrogel samples and the water-swollen samples. The resulting masses were then substituted into eqs 1 and 2.^62^ By transferring the specimens to Petri plates and allowing them to dry at 60 °C for the subsequent 24 h, we meticulously assessed the effectiveness of the drying procedure.
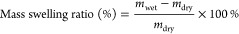
1
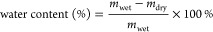
2

In eqs 1 and 2, *m*_wet_ represents the mass of the swollen hydrogel, and *m*_dry_ represents the mass of the dried hydrogel.

### 2.5 Morphology

The synthesized gel samples were analyzed under scanning electron microscopy (SEM) to determine their morphology. Following freeze-drying, the hydrogels were cut into cross sections and examined under a microscope. A Labconco FreeZone lyophilizer was used to freeze-dry the samples at −82 °C and 0.03 mbar pressure, after which they were frozen in liquid nitrogen to preserve their porosity structure. Using a Polaron SC7620 tiny sputter coater, a 3 nm thick layer of sputtered Au–Pd alloy was applied to the samples prior to image capture. A Zeiss Merlin field-emission microscope was used to obtain the SEM pictures.

### 2.6 Mechanical Properties

The hydrogels manufactured were tested for their mechanical properties by utilizing a 20 N load cell and EZ-SX Shim-pol tensile test equipment. Each test was repeated at least three times. The strain rate used to conduct the tensile tests was 50 mm·min^–1^. The gel sample had a cylindrical shape with a diameter of 4 mm and a length of 10 mm.

For compression testing, the hydrogel samples were cylindrical of diameter 10 mm. The height was compressed at a rate of 5 mm·min^–1^ at room temperature.

The rheological tests were carried out using a 15 mm diameter plate-and-plate geometry MCR302 strain-controlled rheometer (Anton Paar, Graz, Austria). Two different measurement methods were used:

1. The amplitude sweep tests were conducted at 20 °C with an oscillatory strain ranging from small (γ = 0.1%) to a large value (γ = 1000%) and a fixed angular frequency of 10 rad·s^–1^.
2. The frequency sweep experiments were performed over a frequency range of 0.1 to 100 rad·s^–1^, with a fixed strain amplitude of 10% (γ). During all rheological experiments, a cap was used to prevent water evaporation and maintain a constant temperature. A PolyScience circulating bath was used for temperature control. Each test was run three times.

### 2.7 Conductivity

To measure the conductivity of the hydrogels, we employed a conductometer (Mettler Toledo, EasyFive). Additionally, a simple circuit was constructed comprising a 1 cm × 1 cm × 0.5 cm segment of the hydrogel, a LED bulb, and two 1.5 V batteries. This circuit served to indicate the existence and restoration of good conductivity within the hydrogel. Glowing of the LED bulb provided visual confirmation of the hydrogel’s conductive properties.

### 2.8 Motion-Sensing Measurements

The response of the sensor hydrogels was evaluated using an electrochemical workstation (CHI950, CH Instruments, USA). First, the current–voltage characteristics were examined. Cyclic voltammograms were obtained to determine the appropriate potential for the measurements. Two potentials were selected. The current through the P(AM_AcOr_Gelatin) and P(AM_AcOr_Gelatin)-Cu^2+^ hydrogels was measured by applying a potential difference of 3 V, while 0.5 V was applied for P(AM_AcOr_Gelatin)-Fe^3+^. A lower voltage was chosen for iron because a Faradaic reaction between the hydrogel and the Al foil occurred at 3 V, which led to a drop of current finally to zero.

Next, the hydrogels were examined for changes in their resistance when bending the layers at different angles (ranging from 30 to 125°) and when subjecting them to various strains (ranging from 50 to 250%). Equation 3 was used to quantify the relative resistance change of the hydrogels.
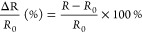
3

*R*_0_ in eq 3 represents the initial resistance of the hydrogels when they are not stretched, and *R* represents the resistance of the hydrogels when they are stretched.^63^

GF, the ratio of fractional change in electrical resistance to the fractional change in length (strain), is the other parameter; it is determined using eq 4
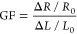
4where *L*_0_ is the initial length and *L* is the length after stretching of the hydrogel.^64^

## 3 Results and Discussion

### 3.1 Preparation and Characterization of the P(AM_AcOr_Gelatin) Hydrogel

**Figure 1:**
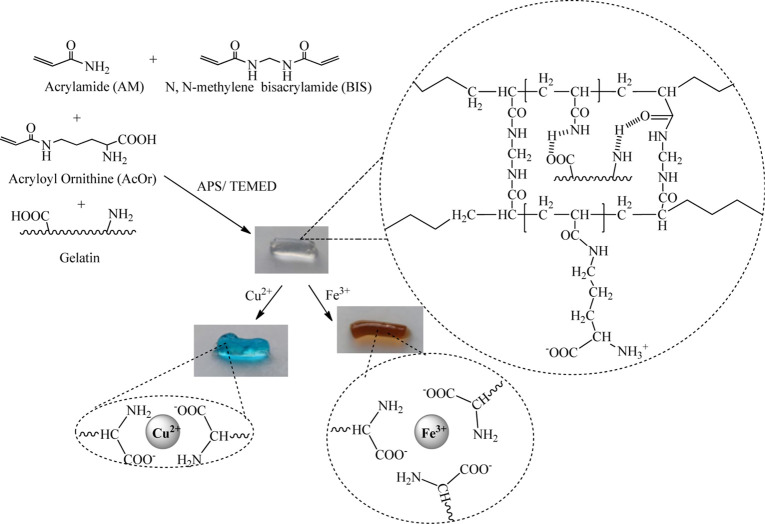
Polymerization scheme and chemical structure of P(AM_AcOr_Gelatin)-Me^n+^ hydrogels.

The aim of this work was to combine a synthetic polymer and a natural polymer to create a hydrogel with mechanical stability and conductivity. We focused on the P(AM_AcOr_Gelatin) hydrogels, where a significant number of noncovalent bonds originating from amino acid-based monomers (AcOr) and gelatin contributed to the enhancement of the mechanical abilities and conductivity of the material. The hydrogels consisting of AM, AcOr, and gelatin were formed through a single-step free-radical polymerization process. It is illustrated in Figure 1. AM, AcOr, and gelatin created physical cross-linking via multiple hydrogen and electrostatic bonds and formed the second network which bolstered the mechanical strength. Moreover, due to the presence of carboxylate groups that are commonly employed as the chelating agents, gelatin and AcOr demonstrated the ability to coordinate metal cations (copper and ferric ions).^65^ As a result, metal ions served as efficient sources of additional cross-linking points and led to the formation of the P(AM_AcOr_Gelatin)-Fe^3+^ and -Cu^2+^ hydrogel variants.

**Figure 2:**
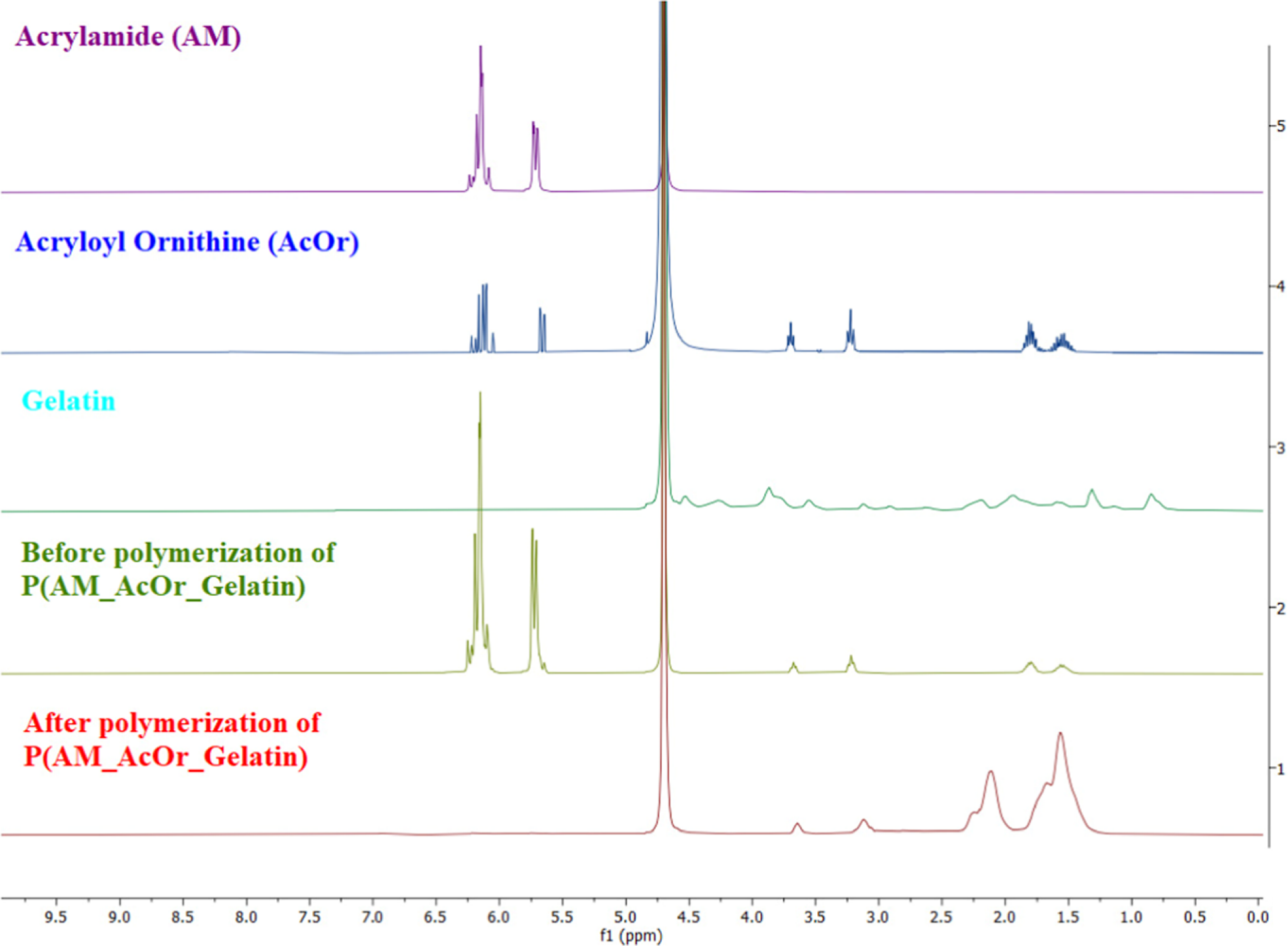
Comparison of ^1^HNMR spectra of acrylamide, acryloyl ornithine monomers, gelatin, and before and after polymerization of hydrogel P(AM_AcOr_Gelatin).

We employed the ^1^HNMR method to characterize the synthesized hydrogel structure. Before the measurements, the AM and AcOr monomers, gelatin, and hydrogel sample were freeze-dried and dissolved in D_2_O. The spectra of the AcOr and AM monomers, gelatin, and hydrogel are presented in Figure 2. The absence of characteristic signals of the monomers at 5.6 and 6.4 ppm was related to the disappearance of the acryl groups after the polymerization. Large signals in the 1.0–2.5 ppm range are attributed to the –(CH_2_–CHR)_*n*_- groups from the polymer network formed by polymerization of AM, AcOr, and BISS. Additionally, all peaks of the AcOr seen in the range 3–4 ppm were still present in the hydrogel spectra; however, at a much lower concentration. They also became broader and partially lost their subtle structure. These ^1^HNMR findings demonstrated successful synthesis of the hydrogel, confirming the presence of AcOr and AM monomers, which underwent complete polymerization.

The peaks originating from gelatin are characterized by low intensity, as can be seen in Figure 2, making them nearly invisible on both ^1^HNMR spectra before and after polymerization of the gel P(AM_AcOr_Gelatin). Therefore, we used the FT-IR technique to prove the presence of gelatin inside the synthesized gel.

Similarly, the difficulty in detecting the peaks of BIS is attributed to small quantity of this cross-linker contained within the gel, which also contributes to its limited visibility on the spectrum.

**Figure 3:**
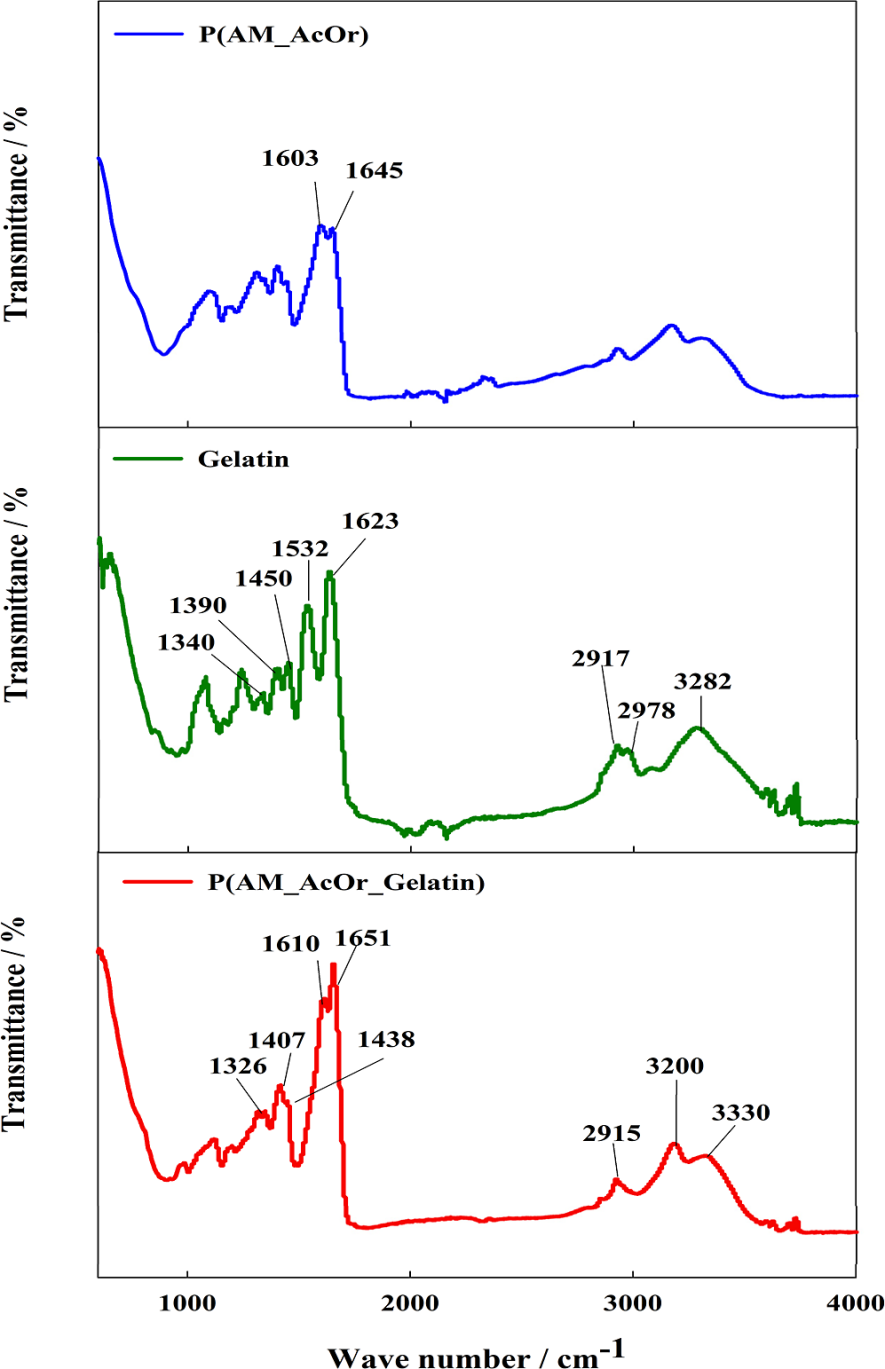
FT-IR spectra of Gelatin and the synthesized hydrogels P(AM_AcOr) and P(AM_AcOr_Gelatin).

Further analysis of the synthesized hydrogel was done with the FT-IR method to confirm the existence of gelatin within the synthesized gel. Figure 3 displays the infrared spectra of gelatin, P(AM_AcOr), and P(AM_AcOr_Gelatin). The gelatin characteristic-group bands that are seen at 3282 cm^–1^ correspond to the NH stretching and the hydrogen bonds. The bands at 2978 and 2917 cm^–1^ are attributed to the CH stretching of the CH_2_ groups. The absorption band that appears at 1623 and 1532 cm^–1^ is related to the CO stretching in the amide I vibration, while the band at 1450 and 1390 cm^–1^ (N–H and C–N) originates from the amide II bending vibration. Additionally, the presence of amide III is seen at 1340 cm^–1^.

The hydrogel spectrum exhibited parallels to the gelatin spectrum, albeit with differences in intensity and small shifts. Notably, characteristic absorptions were identified at 1651 cm^–1^ and 1610 cm^–1^, corresponding to the CO stretching in amide I and NH_2_ deformation, respectively. The P(AM_AcOr) spectrum showed that the ratio of the peaks at 1610 and 1651 cm^–1^ changed substantially after polymerization with gelatin. Bands at 1438 cm^–1^ and 1407 cm^–1^ were assigned to the CH_2_ deformation and the C–N stretching, respectively. Furthermore, a distinct peak at 1326 cm^–1^ attributed to CH deformation was observed. Interestingly, the 1450 cm^–1^ absorption band observed in the gelatin spectrum appeared only as a shoulder in the hydrogel spectrum.^66−68^

### 3.2 Swelling Ratio

The swelling ratio data of the prepared hydrogels are presented in Table 1. Water content in the modified hydrogels containing copper and ferric ions was noticeably reduced. As is evident, P(AM_AcOr_Gelatin) exhibited a mass swelling ratio of 931.3%. It decreased to 768.9% for P(AM_AcOr_Gelatin)-Cu^2+^ and was further reduced to ca. 619.4% for P(AM_AcOr_Gelatin)-Fe^3+^. This decrease in the swelling ratio is explained by the formation of coordination bonds between Fe^3+^ and Cu^2+^ and the polymer net functional groups.^69^

**Table 1:** Water Content and Mass Swelling Ratio of Modified and Unmodified Gel Samples

sample	water content/%	swelling ratio/%
P(AM_AcOr_Gelatin)	90.3	931.3
P(AM_AcOr_Gelatin)-Cu^2+^	88.5	768.9
P(AM_AcOr_Gelatin)-Fe^3+^	86.1	619.4

### 3.3 Surface Morphology

**Figure 4:**
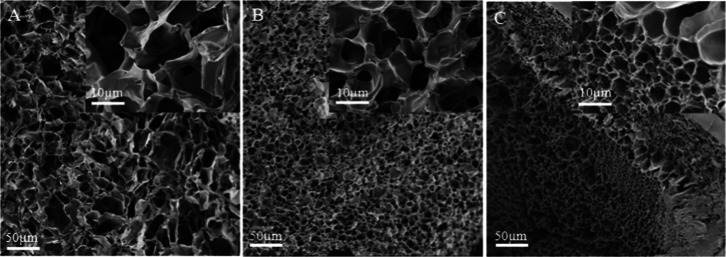
SEM images of: (A) P(AM_AcOr_Gelatin), (B) P(AM_AcOr_Gelatin)-Cu^2+^, and (C) P(AM_AcOr_Gelatin)-Fe^3+^.

SEM was used to examine the shape and structure of the hydrogels. The hydrogel samples were freeze-dried and fractured to characterize the three-dimensional networks in their swelled form. Typical cross-sectional surface pictures of unmodified and metal-ion-modified hydrogels at two different magnifications (500 and 2500) are depicted in Figure 4. Each sample exhibits a typical porous three-dimensional structural network with connected pores as well as a highly interconnected network of gelatin, AcOr, and polyacrylamide. The hydrogel distinctive network structure is a crucial characteristic that greatly influences other attributes.

Figure 4 images demonstrate how the addition of metal ions to the gel structures altered their porosity, average pore size, and pore size distribution. Following the modification by copper and ferric ions, the hydrogel’s pore size dropped from 17 ± 3 to 10 ± 2 and to 5 ± 2 μm, respectively. The reason for this was the interactions between the AcOr molecules with both ions, which macroscopically causes the hydrogel’s internal pore density to increase and its pore size to decrease. Both ArOr and gelatin have functional bidentate groups or ligands that are capable of forming complexes with metal ions with a stoichiometry larger than 1:1. This means that additional cross-linking points may appear in the polymer network. Additionally, the stoichiometries of cupric and ferric ion complexes differ. The copper(II) ions can form complexes with α-amino acids with a maximal stoichiometry of 1:2, while iron(III) can reach 1:3.^56,57^ This explains the higher cross-linking density of iron species as it serves as the coordination center for three chelate groups and optimally links three polymer chains. Certainly, iron species can also be complexed by three groups within the same or two polymer chains.

### 3.4 Investigation of Mechanical Properties

#### 3.4.1 Tensile Test

**Figure 5:**
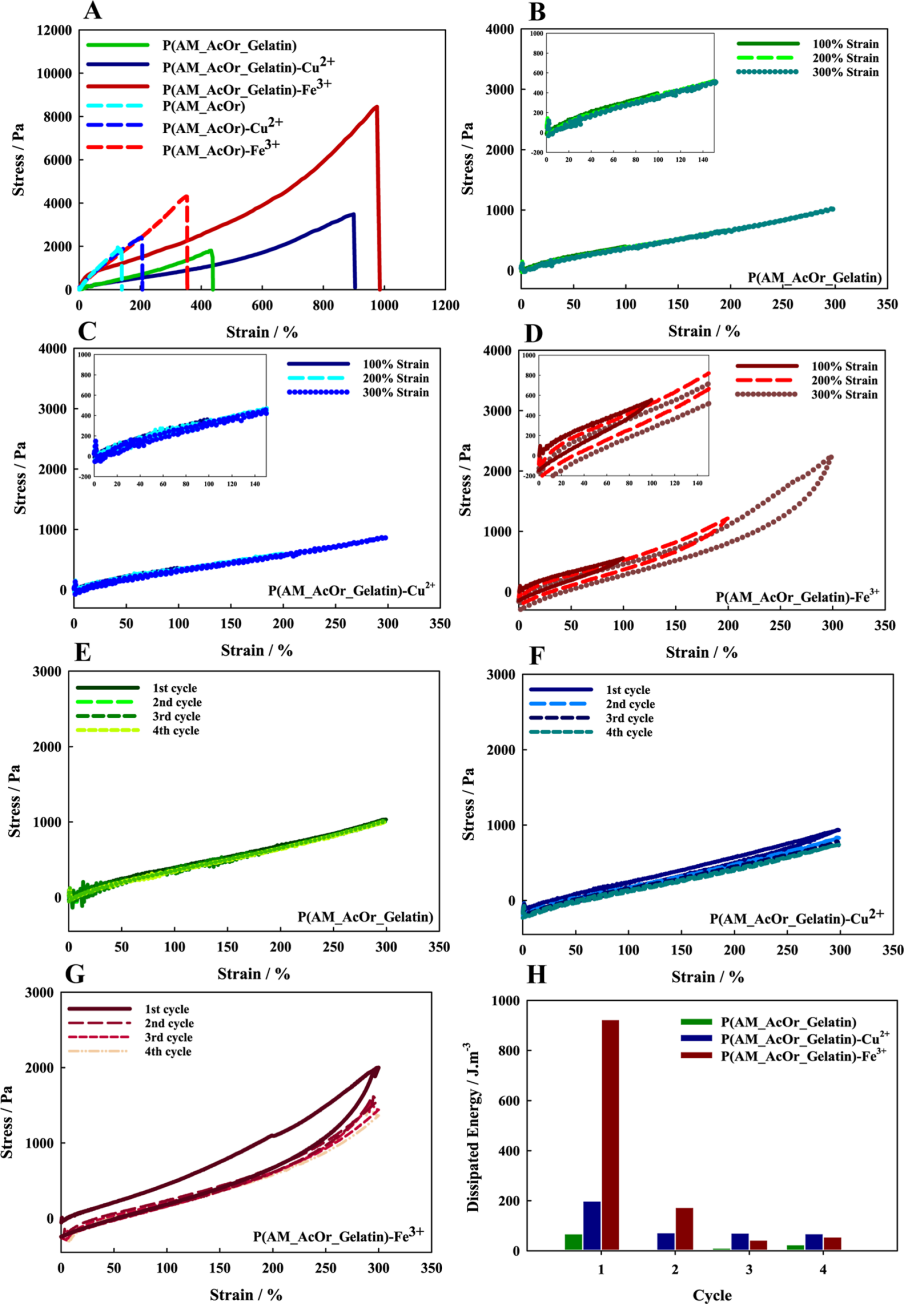
(A) Tensile curves of hydrogels prepared with and without gelatin. Cyclic tensile curves for (B) P(AM_AcOr_Gelatin), (C) P(AM_AcOr_Gelatin)- Cu^2+^, and (D) P(AM_AcOr_Gelatin)-Fe^3+^ under various strains (100–300%). Fatigue curves for (E) P(AM_AcOr_Gelatin), (F) P(AM_AcOr_Gelatin)-Cu^2+^, and (G) P(AM_AcOr_Gelatin)-Fe^3+^ under 300% strain for 4 cycles. (H) Energy dissipation in consecutive cycles under 300% tensile strain for prepared hydrogel samples.

A universal tensile machine was used to characterize the mechanical properties of the hydrogels. Figure 5 presents the obtained data. Using cyclic and conventional tensile tests, we have examined the mechanical characteristics of three hydrogels: P(AM_AcOr_Gelatin), P(AM_AcOr_Gelatin)-Fe^3+^, and P(AM_AcOr_Gelatin)-Cu^2+^.

Typical stress–strain curves of the prepared hydrogels are shown in Figure 5A. The P(AM_AcOr_Gelatin)-Cu^2+^ gel could be extended ca. 9 times its original length without rupture, with a rupture strength of 3500 ± 625 Pa at 900 ± 93% strain. The P(AM_AcOr_Gelatin)-Fe^3+^ hydrogel could be extended 10 times (1000% ± 128) with a much higher stress strength of 8400 ± 937 Pa. However, the P(AM_AcOr_Gelatin) hydrogel could only be stretched to 4.5 times (434 ± 150%) its original length without rupture with a strength of 1400 ± 563 Pa. The stretchability and strength were both associated with the complex formation in the polymer network, which increased the cross-linking density in the hydrogels.

Furthermore, the addition of gelatin as a second network enhanced P(AM_AcOr)’s mechanical stretchability, which without gelatin was broken at 133 ± 45% (stress of 1843 ± 342 Pa). Additionally, P(AM_AcOr)-Cu^2+^ and P(AM_AcOr)-Fe^3+^ showed elongations at a break of 206 ± 68% (stress of 2398 ± 434 Pa) and 353 ± 105% (stress of 4301 ± 576 Pa), respectively. To conclude, the introduction of metal ions and gelatin improved both the toughness and stretchability of the gels.

To further investigate the behavior of the hydrogel network upon strain, we performed the loading–unloading cyclic test. First, tests with increasing strain on all three hydrogels (100–300% for cyclic tensile) were performed, see Figure 5B–D. The P(AM_AcOr_Gelatin) and P(AM_AcOr_Gelatin)-Cu^2+^ hydrogels exhibited elastic behavior; almost no hysteresis was observed. In this case, negligible hysteresis could be related to fast recovery of electrostatic interactions and hydrogen bonds between gelatin and the p(AM_AcOr) network, and elastic behavior is observed. For P(AM_AcOr_Gelatin)-Fe^3+^, “Mullin’s effect” is observed: stretching–relaxation cycles show hysteresis even at a small strain. In this case, the recovery of the coordination bond between iron ions and carboxylic groups caused a rise of the hysteresis loop; also, some residual strain is observed. The additional cyclic loading–unloading tests to constant 300% strain were performed to check the ability of the polymer network to dissipate energy, see Figure 5E–G. For all samples, the 300% level is below the critical strain. The P(AM_AcOr_Gelatin) gel responded elastically, and each cycle followed the first load curve. For the hydrogel with coordinated metal ions, the hysteresis loop appeared in the first cycle, which is especially well visible in the case of iron ions. The P(AM_AcOr_Gelatin)-Fe^3+^ response was typical for the hybrid cross-linked materials, where in the first cycle of loading–unloading, the higher amount of energy is dissipated and, in further steps, the dissipation is insignificant and the cycles are nearly elastic, see Figure 5G. For P(AM_AcOr_Gelatin) and P(AM_AcOr_Gelatin)-Cu^2+^ for applied elongations, the effect was less significant, the dissipation energies were small, and the responses were close to the elastic one. Regarding the hysteresis loop for gel containing Fe^3+^, a higher energy loss is observed because more cross-linking bonds are destructed during the deformation. While copper and iron complexes are comparably thermodynamically stable, based on the stability constants,^70,71^ the observed behavior—hysteresis—may origin from different kinetics and different cross-link densities due to different complex stoichiometries. Iron ions create also complexes with higher stoichiometry, forming more interactions with chains, which leads to higher hysteresis and smaller resilience. Additionally, for P(AM_AcOr_Gelatin) and P(AM_AcOr_Gelatin)-Cu^2+^, there was no residual stress observed, which means that the plastic deformations did not occur, and it is relevant in terms of using this material as a skin motion sensor. As shown in Figure 5E–G, the loading–unloading curves for all samples in the second to fourth cycles exhibited minor variations. These graphs revealed the good fatigue resistance of the hydrogels, as evidenced by nearly overlapping hysteresis loops.

#### 3.4.2 Compression Test

**Figure 6:**
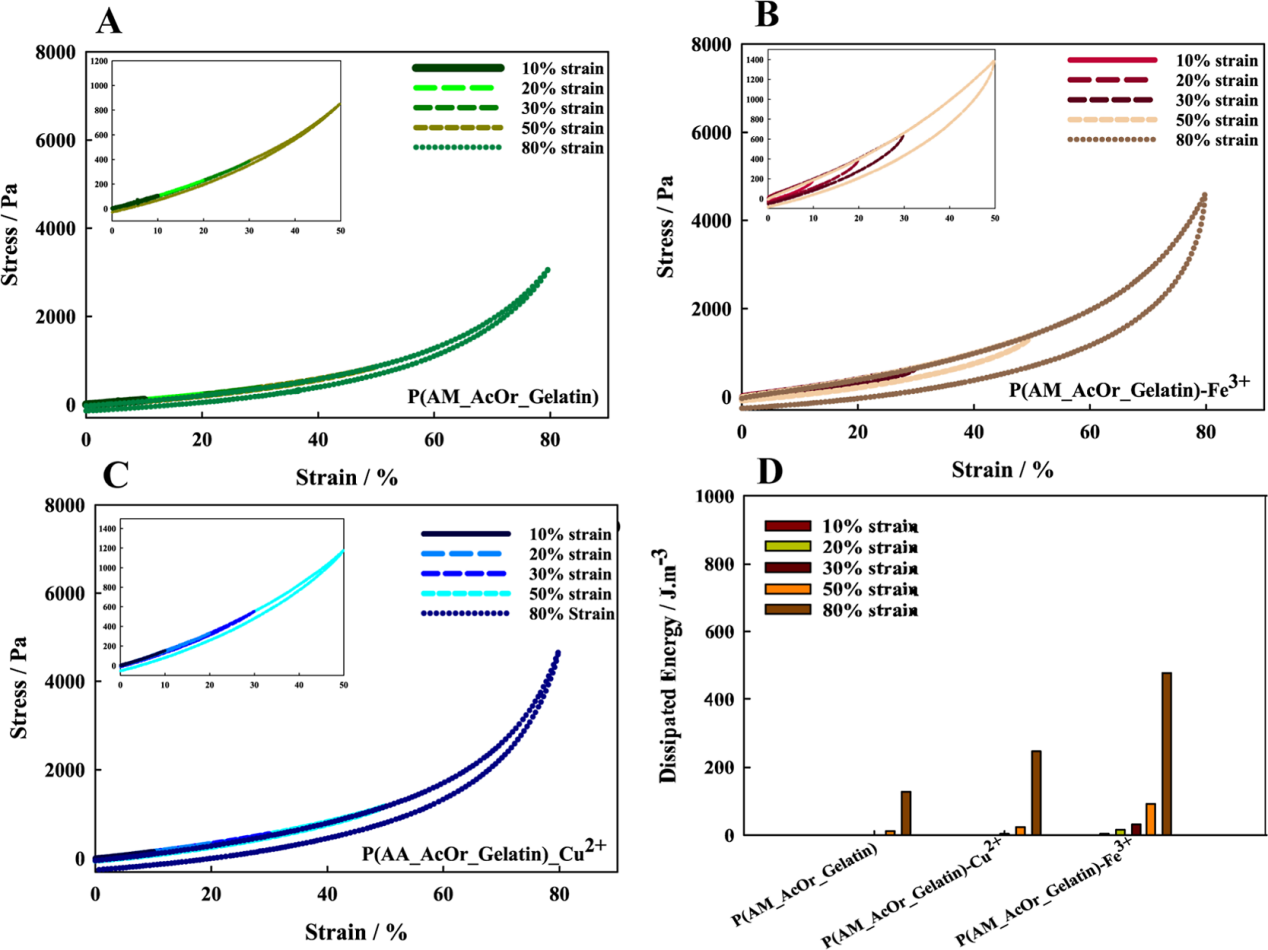
Cyclic compression curves of (A) P(AM_AcOr_Gelatin), (B) P(AM_AcOr_Gelatin)-Cu^2+^, and (C) P(AM_AcOr_Gelatin)-Fe^3+^ under various strains (from 10% to 80%). (D) Energy dissipation (bars) under different compression strains (from 10% to 80%) for all prepared samples.

To assess the hydrogel ability of compression recovery, more loading–unloading cycles under increasing strain (10–80%) were performed with the hydrogels. The loading–unloading compression measurements were consistent with those of the cyclic loading–unloading tensile tests. As it is shown in Figure 6A,B, for P(AM_AcOr_Gelatin) and P(AM_AcOr_Gelatin)-Cu^2+^, the compression unloading curve followed the loading curve up to the 50% deformation, and it confirmed elastic-like behavior of the materials. Small hysteresis loops were observed only for a relatively high compression of 80%. Contrarily, for P(AM_AcOr_Gelatin)-Fe^3+^, the hysteresis loops appeared even for small compressions, see Figure 6C. The values of dissipated energies for various compressions are presented in Figure 6D.

The synthesized ion-modified hydrogels demonstrated improved tensile/compressive strength as well as good durability and elasticity (especially in the case of P(AM AcOr Gelatin)-Cu^2+^) after cyclic loading and unloading, as indicated by the above-mentioned results. Sufficiently high mechanical strength is essential for the strain-sensor construction.

#### 3.4.3 Rheometer Test

The rheological technique was used to comprehensively assess the internal structure and mechanical properties of the hydrogels. Parameters such as the storage modulus (*G*′) and loss modulus (*G*″), indicative of elasticity and viscosity, respectively, were evaluated to understand the behavior of hydrogels under various conditions such as shear stress, frequency, and time.

**Figure 7:**
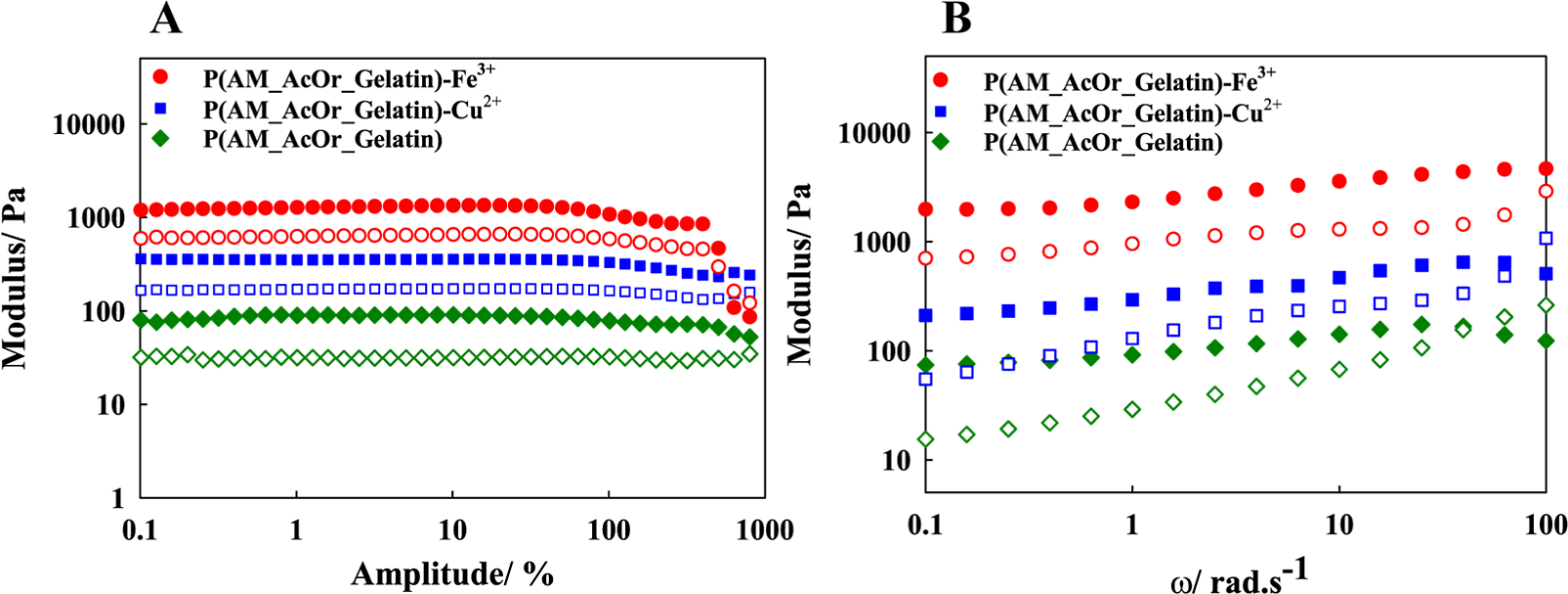
(A) Amplitude sweep test and (B) frequency sweep test of P(AM_AcOr_Gelatin), P(AM_AcOr_Gelatin)-Cu^2+^, and P(AM_AcOr_Gelatin)-Fe^3+^. Storage modulus and loss modulus are shown as filled symbols and unfilled symbols, respectively.

In this study, the microstructures of the synthesized hydrogel series were thoroughly investigated through rheological measurements. Figure 7A illustrates the changes in storage and loss moduli with amplitude (γ), revealing that for all hydrogels, a rather wide linear viscoelastic region existed. A shear amplitude of γ = 10% was selected for subsequent frequency sweep measurements. The *G*′ and *G*″ increase in the order P(AM_AcOr_Gelatin) < P(AM_AcOr_Gelatin)-Cu^2+^ < P(AM_AcOr_Gelatin)-Fe^3+^. The introduction of metal ions provides extra cross-linking points which makes the gel structure more robust/stiff. The stoichiometry of iron complexes is higher than that of copper; therefore, the created network is more densely cross-linked, which leads to an increase in both elastic *G*′ and viscous modulus *G*″. Higher *G*″ proves also an increase in capability of energy dissipation by internal friction. The decrease in the critical strain or crossover point for P(AM_AcOr_Gelatin)-Fe^3+^ confirmed that this double network has higher cross-linking density and collapsed at lower amplitudes. The appearance of crossover points means rupture of the gel structure, and gradual decrease of *G*″ indicates gradual breakdown of the structure.

Figure 7B depicts the variation of *G*′ and *G*″ with angular frequencies, demonstrating typical gel-like behavior in the hydrogel samples, with both moduli changing with frequency. This discrepancy suggests that at higher frequencies, where bond formation occurred more rapidly than bond rupture, the energy was swiftly dissipated through dynamic bond reformation. Consequently, the materials exhibited more viscous behavior and were characterized by unrestricted movement of the polymer chains during short periods of deformation.

### 3.5 Conductivity Measurements

**Figure 8:**
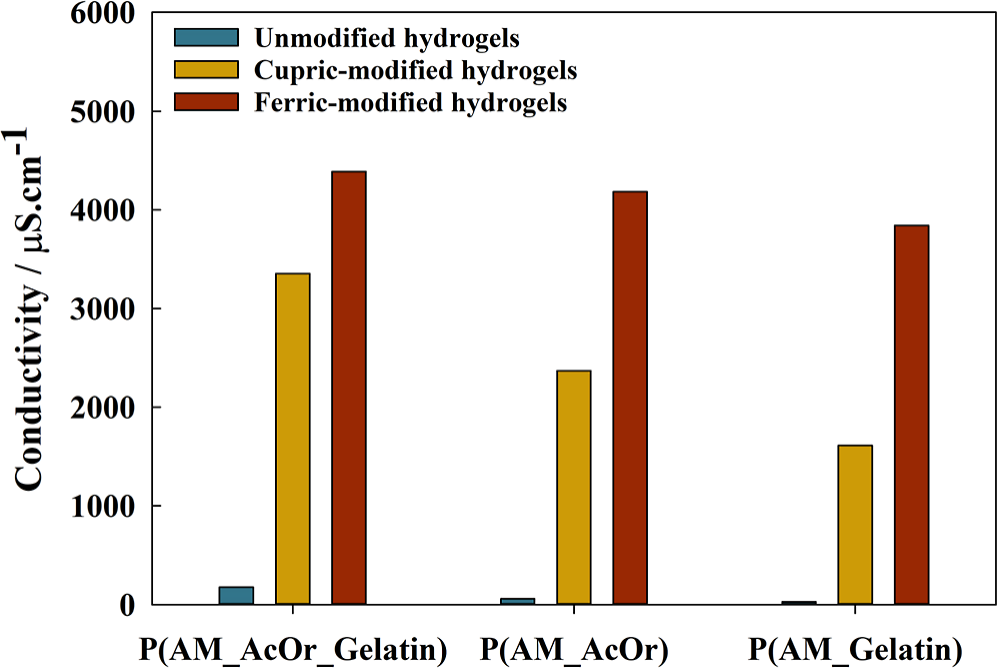
Conductivity of hydrogel samples of different compositions.

Figure 8 displays distinct conductivity values obtained for pure, cupric-, and ferric-modified hydrogels with different compositions. The ions were added to the hydrogels to give good electrical conductivity. As we can see, both introduction of AcOr and metal ions increased the conductivity of the hydrogels. P(AM_AcOr_Gelatin)-Fe^3+^ and -Cu^2+^ had the highest conductivity equal to 4387 and 3355 μS·cm^–1^ (several times higher than that of P(AM_AcOr_Gelatin)). These results indicated their remarkable potential application in the field of soft electronic materials and wearable sensors.^72−74^ The electrical conductivity of ion-modified hydrogels originated from the diffusion of a large number of free counterions and the exchange of electrons between the iron ions (Fe^3+^/Fe^2+^) and copper ions (Cu^2+^/Cu^+^) in the matrices.

**Figure 9:**
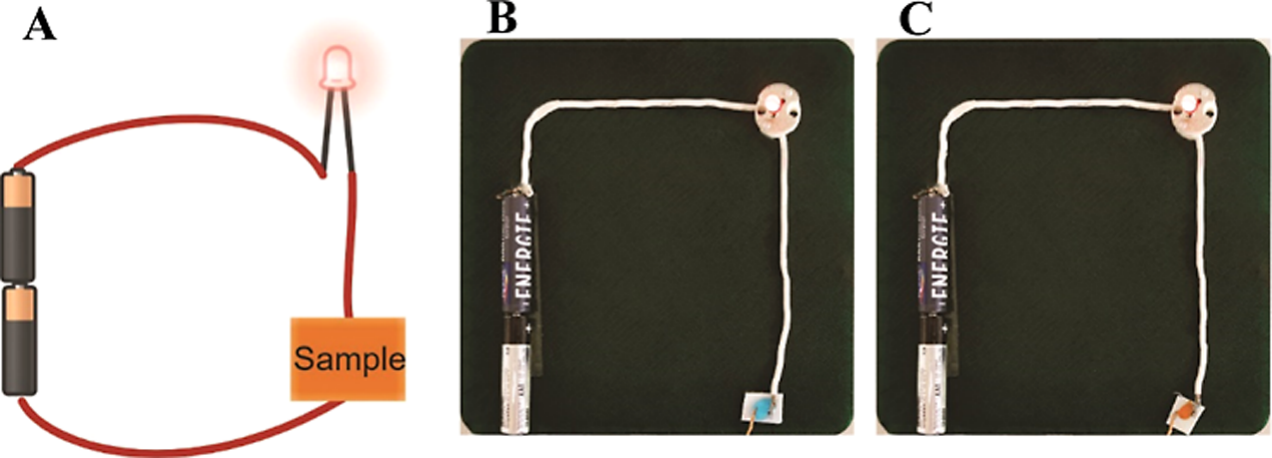
(A) Scheme of a simple electric current indicator. (B,C) Electric circuits for measuring the conductivity/resistance of P(AM_AcOr_Gelatin)-Cu^2+^ and P(AM_AcOr_Gelatin)-Fe^3+^, respectively.

To demonstrate the conductivity of the modified hydrogel samples, a simple electric circuit shown in Figure 9 was assembled. When the electric circuit was broken, the light was off, and the addition of the gel sample of either P(AM_AcOr_Gelatin)-Cu^2+^ or P(AM_AcOr_Gelatin)-Fe^3+^ completed the circuit and turned the light up.

### 3.6 Strain-Sensing Behavior

**Figure 10:**
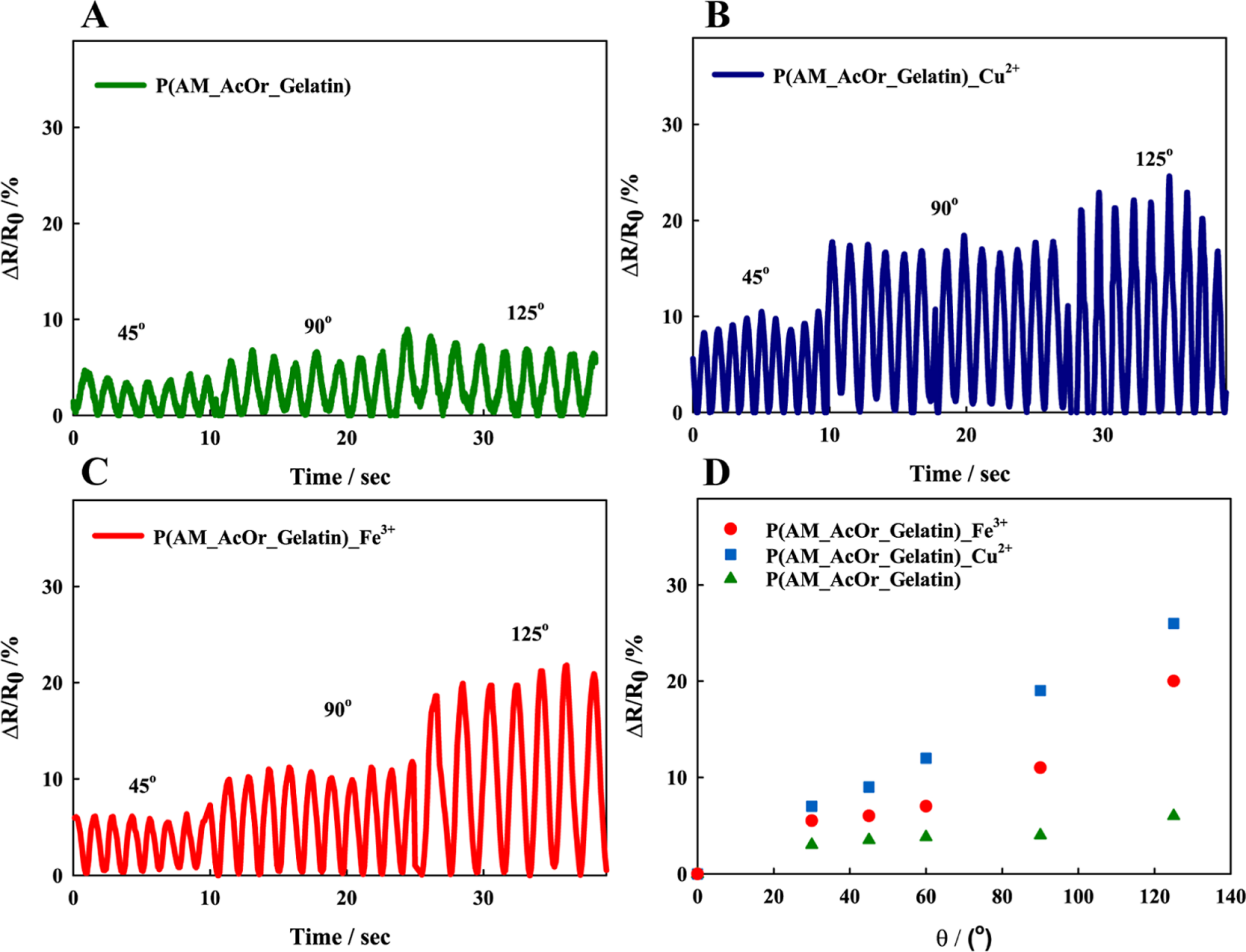
Relative resistance versus time for different bending angles for (A) P(AM_AcOr_Gelatin), (B) P(AM_AcOr_Gelatin)-Cu^2+^, and (C) P(AM_AcOr_Gelatin)-Fe^3+^. (D) Relative resistance plotted versus the bending angle for P(AM_AcOr_Gelatin), P(AM_AcOr_Gelatin)-Cu^2+^, and P(AM_AcOr_Gelatin)-Fe^3+^.

**Figure 11:**
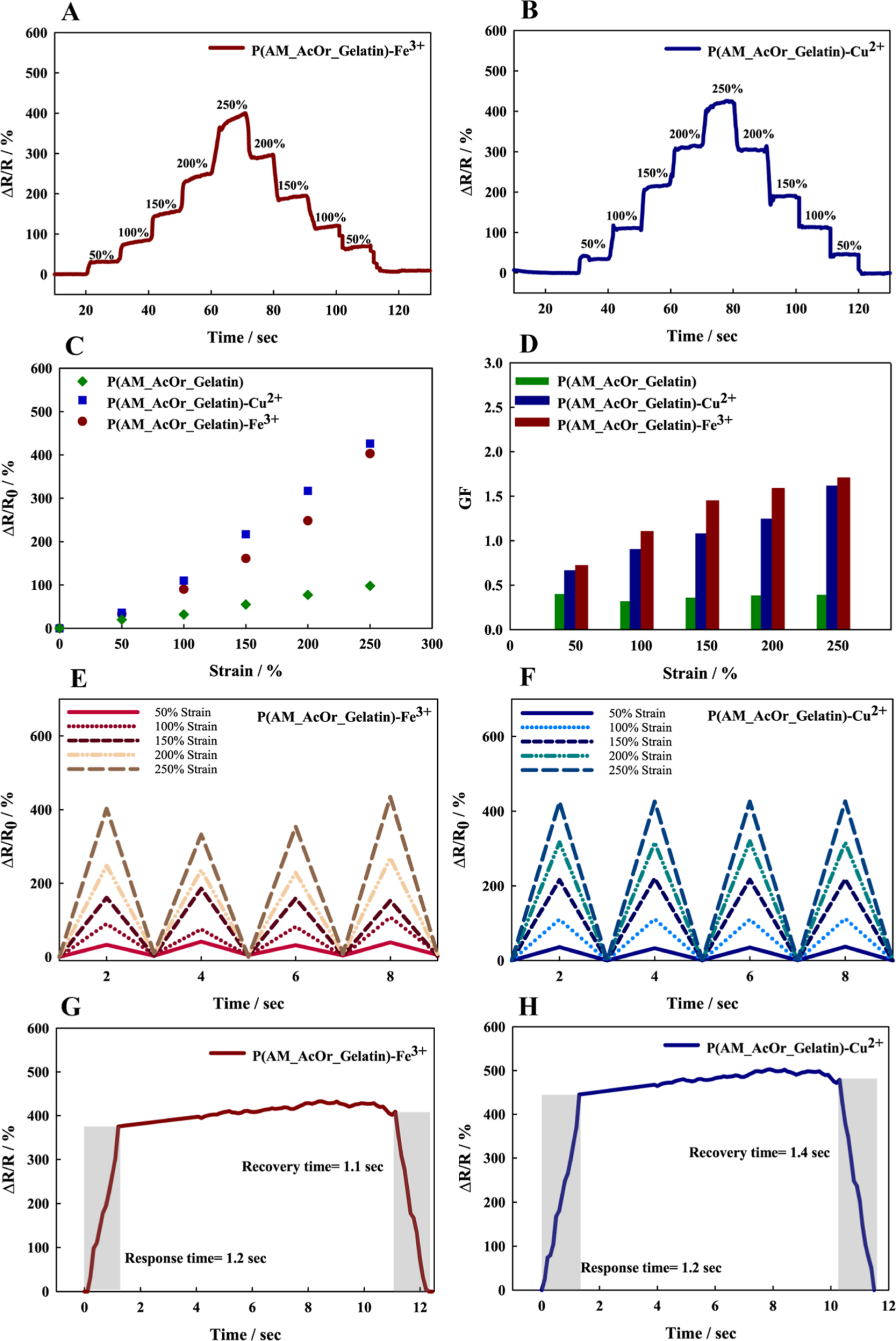
Relative resistance versus time in hydrogels under different strains (50–250%) for (A) P(AM_AcOr_Gelatin)- Fe^3+^ and (B) P(AM_AcOr_Gelatin)-Cu^2+^. (C) Comparison of relative resistance and (D) GF changes for P(AM_AcOr_Gelatin), P(AM_AcOr_Gelatin)-Cu^2+^, and P(AM_AcOr_Gelatin)-Fe^3+^. Relative resistance versus time under different strains (50–250%) for (E) P(AM_AcOr_Gelatin)- Fe^3+^ and (F) P(AM_AcOr_Gelatin)-Cu^2+^. Response and recovery time for (G) P(AM_AcOr_Gelatin)-Fe^3+^ and (H) P(AM_AcOr_Gelatin)-Cu^2+^.

Conductive hydrogels with appropriate mechanical characteristics emerged as promising options for flexible electronic sensors. To investigate the strain-sensing behavior of the prepared hydrogel-based sensors, first, after analysis of the voltammetric curves, we have chosen a potential for the current measurements. Current was measured versus time under conditions of various strains and finger-bending angles using the chronoamperometric method.^75,76^ Relative resistance, according to eq 3, was calculated and studied for all prepared samples and bending angles (see Figure 10) and for various elongations (see Figure 11). Figure 10 illustrates the real-time sensing changes of the hydrogel integrated with a finger. The magnitudes of relative resistances for metal ion-modified and unmodified P(AM_AcOr_Gelatin) hydrogels were investigated using finger-bending angles of 45, 90, and 125°. For P(AM_AcOr_Gelatin), the relative resistance did not change significantly with an increasing bending angle; see Figure 10A. This was related to poor conductivity of that material, whereas for P(AM_AcOr_Gelatin)-Cu^2+^ and -Fe^3+^, a significant increase in resistance upon bending increase was observed, see Figure 10B,C, respectively. When the hydrogels were bent, their geometric changes and related ion movement obstructions resulted in a meaningful increase in the relative resistance. Because the hydrogel was deformed to varying degrees, the ion-modified P(AM_AcOr_Gelatin) hydrogel sensors were able to easily distinguish between the different bending tendencies. In Figure 10D, the calibration curves for different bending angles of 30, 45, 60, 90, and 125° were prepared. P(AM_AcOr_Gelatin)-Fe^3+^ and -Cu^2+^ showed excellent bending sensitivity. These hydrogels effectively converted the deformation into an electric signal change with good linear response. The poor sensitivity was observed only for P(AM_AcOr_Gelatin). Therefore, for further experiments, the ion-modified gels were used.

Figure 11A,B presents relative resistance changes after an increase in elongation of the hydrogel sample by 50%. The hydrogels were equilibrated for 10 s after each 50% stretching. Then the entire response and recovery process was observed. As can be seen for P(AM_AcOr_Gelatin)-Cu^2+^, the obtained stairs/steps were symmetrical and there was no drift in relative resistance at any step, whereas for P(AM_AcOr_Gelatin)-Fe^3+^, the response was rather asymmetrical, the relative resistance value depended on the direction of the applied force and did not have the same value for the measured strains. It can be explained on the basis of mechanical properties of the material. The cyclic strain analysis of the iron-modified gel showed hysteresis, which may explain the material’s sensing response. Also, drifts were observed at most of the steps. The relative resistance and GF versus strain in the range of 0–250% are shown in Figure 11C and D, respectively. The copper-modified hydrogel exhibited higher sensitivity to strain change than the ferric-modified hydrogel-based sensor (Figure 11C).

The GF, a critical parameter indicating sensor sensitivity, was calculated from the slope of the Δ*R*/*R*_0_ versus strain curve. Figure 11D illustrates that the GFs of the hydrogels exhibited an increase with increasing strain, ranging from 50% to 250%. Within this strain range, the GF values were in the range from 0 to 1.7, signifying very good sensor sensitivity.

The sensor performance stabilities of the hydrogels under elongations ranging from 50 to 250% are depicted in Figure 11E,F. Notably, the hydrogels modified with copper demonstrated exceptional stability across various elongations. However, for hydrogels modified with the ferric ions, a wider spread of results was observed. Despite this variability, the overall stability of the ferric-modified hydrogels remained reasonable across the tested elongation range. Additionally, based on Figure 11G,H, the response times of both hydrogels are similar (1.2 s). However, the recovery time for copper is slightly longer (by 0.3 s).

## 4 Conclusions

In this study, we successfully synthesized double-network P(AM_AcOr_Gelatin) hydrogels and modified them with metal ions (cupric and ferric ions). These hydrogels modified with metal ions, P(AM_AcOr_Gelatin)-Cu^2+^ and P(AM_AcOr_Gelatin)-Fe^3+^, formed electrostatic cross-linked structures as a result of interactions between the AcOr monomer and metal ions. As a result, they obtained improved mechanical properties, increased conductivity, and remarkable motion sensitivity. The detection of motion amplitude was possible by measuring the constant-potential current flowing through the sensor hydrogel layer. The current intensity was related to actual conductivity and resistance of the gel, which were dependent on its bending angle. Interestingly, the hydrogel modified with cupric ion showed a more consistent sensing behavior compared to the hydrogel modified with ferric ion, which was due to its inherently more elastic structure. However, the gel modified with ferric ions showed higher toughness and stretchability.

With their remarkable characteristics, these hydrogels show great potential in the field of materials for wearable motion sensors that are subjected to significant deformations. The unique combination of flexibility, stability, and responsiveness makes them ideal for applications that require durable yet adaptable sensor materials in wearable technology.

## Data Availability

The data underlying this study are available throughout the manuscript.
